# Robust estimation of the latent trait in graded response models

**DOI:** 10.3758/s13428-024-02574-2

**Published:** 2025-01-14

**Authors:** Audrey Filonczuk, Ying Cheng

**Affiliations:** Department of Psychology, University of Notre Dame, 390 Corbett Hall, Notre Dame, IN 46556 USA

**Keywords:** Robust estimation, Graded response model (GRM), Item response theory, Aberrant responses

## Abstract

Aberrant responses (e.g., careless responses, miskeyed items, etc.) often contaminate psychological assessments and surveys. Previous robust estimators for dichotomous IRT models have produced more accurate latent trait estimates with data containing response disturbances. However, for widely used Likert-type items with three or more response categories, a robust estimator for estimating latent traits does not exist. We propose a robust estimator for the graded response model (GRM) that can be applied to Likert-type items. Two weighting mechanisms for downweighting “suspicious” responses are considered: the Huber and the bisquare weight functions. Simulations reveal the estimator reduces bias for various test lengths, numbers of response categories, and types of response disturbances. The reduction in bias and stable standard errors suggests that the robust estimator for the GRM is effective in counteracting the harmful effects of response disturbances and providing more accurate scores on psychological assessments. The robust estimator is then applied to data from the Big Five Inventory-2 (Ober et al., 2021) to demonstrate its use. Potential applications and implications are discussed.

## Introduction

Likert-type items are commonly used in psychological and educational assessments (exams, tests, surveys, etc.). Item response theory (IRT) models such as the graded response model (GRM) have been developed to model the probability that a subject responds in a certain category on these items (Samejima, 1969). Given a subject’s response vector and the item parameters, one can estimate their latent trait, $\theta ,$ via methods such as maximum likelihood (ML) estimation. However, responses to these items may not follow the assumed IRT model if the respondent exhibits one or more types of aberrant behaviors.

In the context of high-stakes testing, a variety of aberrant behaviors can plague a subject’s responses on such assessments, such as the six examples outlined by Meijer (1996). A subject may exhibit *sleeping* or *warm up* behavior, giving less effort to or being less focused on early items but paying greater attention to items appearing later in the test. *Guessing* occurs when a subject of low ability guesses blindly to items of higher difficulty or items towards the end of an assessment under time pressure. *Cheating* can occur when a subject with low ability copies answers from a subject of high ability. *Plodding* describes a subject who responds very slowly and carefully to each item. *Alignment errors* may occur when the response on the answer sheet does not match the intended response. Finally, some subjects of high ability may be *extremely creative,* reinterpreting easy items and responding incorrectly to them. In the low-stakes assessment context, aberrant behaviors such as careless or insufficient effort (C/IE) responding have also received a lot of attention, in which the subject responds with reduced attention to the item (Curran, 2016; Hong et al., 2020). C/IE responding can be *nonrandom* (e.g., endorsing the same response category) or *random* (Meade & Craig, 2012).

When a response vector contains even just some aberrant responses, traditional methods such as ML estimation result in biased estimates of the latent trait (De Ayala et al., 2000; Mislevy & Bock, 1982). Spuriously high or spuriously low assessment scores may yield undesirable consequences, such as giving opportunities to subjects who are not qualified, or failing to identify subjects who need further attention in clinical settings. Beyond latent trait estimates, other statistical properties of the test may be compromised, such as decreased scale reliability (Huang et al., 2012), poor factor model fit (Woods, 2006), lower predictive validity (Hong & Cheng, 2019a), and biased structural parameters (Oshima, 1994; Wise et al., 2004). If the aberrant subjects are known, steps can be taken (e.g., retesting or removing these subjects from the data) in order to eliminate the bias produced by the response disturbances. However, the identity of aberrant subjects is often unknown. Therefore, in order to minimize the bias, it is important to detect aberrant responses and then reduce or eliminate their negative impact.

Several methods have been developed in order to address aberrant responses to polytomous items. However, much of the previous literature focuses on fully removing cases marked as aberrant. Given the controversy of data removal, and to avoid losing information, we seek to downweight responses according to their degree of aberrancy instead of removing them. Hence, a robust estimator for the GRM will be derived to obtain more accurate latent trait estimates. With two examples of aberrant behavior, we aim to demonstrate that these robust estimates are less biased than the maximum likelihood estimates (MLE) in the presence of response disturbances.

### Statistical methods to handle response disturbances

In the IRT literature, which focuses more on achievement or ability testing, a variety of methods have been developed as well to address the issue of aberrant responses. These methods generally handle response disturbances in one of two ways: modeling aberrant behavior or detecting aberrant behavior. In the former approach, traditional IRT models (e.g., the 1-PL, 2-PL, and 3-PL IRT models) are replaced with other models that accommodate the aberrant behavior. For example, speededness may be modeled as a reduction in ability, either abruptly (Yamamoto, 1982, 1995) or gradually (Goegebeur et al., 2008). Or, the dimension representing ability may be accompanied by another dimension to represent the aberrant behavior, such as an auxiliary dimension to represent speededness (Van Der Linden et al., 1999; Van Der Linden & Xiong, 2013), or an additional factor representing careless response styles (Wetzel & Carstensen, 2017). In some cases, careless response behaviors have been modeled through a sequential decision-making process (Böckenholt, 2017) and flexible item parameter estimates (Falk & Cai, 2016). When the data contain both normal and aberrant responses, mixture models can be used to account for a combination of “solution” or normal response behavior and aberrant response behaviors, including speeding (Bolt et al., 2002; Wang & Xu, 2015) or cheating (Wang et al., 2018).

However, modeling aberrant behaviors requires the researcher to know which aberrant behavior occurs in the data and by the mechanism in which it manifests, an unrealistic assumption in practice since the nature of the aberrant response is rarely known (Meade & Craig, 2012). On the other hand, if the solution or normal behavior is known, any type of behavior that deviate from the known pattern can be detected, regardless of the type or mechanism. This process is the approach to “detect” instead of “model” aberrant responses. Many statistical methods have been proposed to detect potential response disturbances in low-stakes assessments. For instance, Mahalanobis distance can detect aberrant response vectors whose patterns deviate from the centroid of data (Mahalanobis, 1936). Longstring analysis identifies individuals with the longest consecutive string of the same response, repeatedly endorsed (Huang et al., 2012; Costa Jr. & McCrae, 2008; Johnson, 2005). Previous articles provide a thorough review of best practices for using these and other methods (e.g., intra-individual response variability, psychometric synonyms, and psychometric antonyms) of aberrant response detection (Curran, 2016; Hong et al., 2020; Meade & Craig, 2012; Niessen et al., 2016). These methods typically do not involve latent variable modeling.

There are also many statistical methods to detect aberrant responses that are based on a latent variable model and residuals, which quantify how responses deviate from the main model for normal or solution behavior. Such residuals include person-fit statistics that are used to detect misfit between a subject’s test performance and their true latent trait. Typically, person-fit statistics compare an observed item response with an expected item response for a test item (Meijer & Sijtsma, 2001). Various aberrant behaviors can be identified, given that they deviate from the expected behavior of the subject according to the assumed model. Working under the IRT framework, we can leverage the item properties by considering the “appropriateness” of the subject response respective to the IRT model. A person-fit statistic that indicates that the response is atypical given the IRT model suggests that the response may be aberrant. A variety of person-fit statistics have been developed to detect unusual response vectors, such as appropriateness measures (e.g., $Z_3$ and *M*), residual-based statistics (e.g., *U* and *W*), and standardized extended caution indices such as *ECI*1 and *ECI*2 (Drasgow et al., 1985, 1987, 1991; Levine & Rubin, 1979; Levine & Drasgow, 1988; Molenaar & Hoijtink, 1990; Tatsuoka, 1984; Trabin & Weiss, 1983; Wright & Stone, 1979; Wright & Masters, 1982). Karabatsos (2003) demonstrates the relative power of these and other person-fit statistics in detecting various aberrant responses across different test designs. A commonly used IRT-based person-fit statistic is $\ell _z,$ which compares the standardized log-likelihood of a response vector against the standard normal distribution (Drasgow et al., 1985). Previous studies have shown the promising ability of $\ell _z$ to detect C/IE and other types of aberrant responses (Hong et al., 2020; Meijer & Sijtsma, 2001; Niessen et al., 2016).

Responses that are flagged as aberrant by these statistical methods are often removed from the data set. Fully removing cases of aberrant data has been practiced for many decades. For instance, Cronbach (1950) argues that full removal is more appropriate than considering the aberrant responses as valid as other responses. Full removal continues to be a conventional albeit controversial method for handling response disturbances (Hong et al., 2020; Meijer & Sijtsma, 2001).

More recently, other methods stemming from the statistical quality control literature have also been developed to detect aberrant response patterns, such as change-point analyses (CPA) and cumulative sum (CUSUM) chart. In addition to indicating the presence of aberrant responses, these methods also try to determine when a certain response pattern starts deviating from the expected response pattern (Shao et al., 2016; Sinharay, 2016). For example, one CPA procedure detects if back random responding (BRR, which refers to random responding towards the end of an assessment, potentially due to fatigue or loss of interest) occurs and, if yes, when it begins (Yu & Cheng, 2019). Compared to the aforementioned methods, CPA enables partial removal of data, instead of complete removal. For example, when BRR is detected and a change point is identified, all responses after the change point would be excluded from the latent trait estimation.

Likewise, CUSUM procedures, proposed by Page (1954), identify fluctuations in the mean of the variable of interest. Instead of identifying a sharp change in responding during a test, CUSUM can detect a change in the quality of responses based on gradual shifts in the estimated latent trait. For instance, Meijer (2002) demonstrates, with an empirical certification test, how a positive CUSUM trend can indicate “warm-up” behavior (i.e., the respondent answers more items correctly as the test continues) while a negative CUSUM trend can indicate fatigue or guessing due to time constraints. Yu and Cheng (2022) outline a number of CUSUM statistics used to detect aberrant response patterns such as those developed by Armstrong and Shi (2009), Bradlow et al. (1998) and Van Krimpen-Stoop and Meijer (2000, 2001).

However, both CPA and CUSUM procedures still make a strong assumption about respondent behavior and are inflexible. For instance, a respondent could resort to random responding when they encounter a set of challenging questions but later returns to normal responding. Removing all responses beyond a certain point would not be appropriate in that case.

Robust estimation is a procedure where all data are leveraged to the extent possible while counteracting aberrant behavior. The type of robust estimation applied in this paper follows Huber’s M-estimation, a procedure in which data points further away from the centroid of the data receive a smaller weight (Huber, 1981). Accordingly, extreme cases have a smaller impact.

The idea of robust estimation has been applied to counteract aberrant responses. An advantage to using robust estimation is that it downweights data according to the *degree* to which each observation is aberrant. A more “suspicious” observation receives less weight, such that it will contribute less to the overall parameter estimation than a less “suspicious” observation. Thus, robust estimation accounts for partially aberrant responses, instead of removing data according to a binary standard. In the context of latent variable modeling, robust estimation has been employed in a number of applications. For example, in the context of IRT models, Hong and Cheng (2019b) proposed a robust marginal maximum likelihood estimator for estimating *item* parameters (or structural parameters) for Likert-scale items that follow the GRM.

This paper will focus on the maximum likelihood estimate (MLE) of the latent trait in the IRT framework. Some approaches to counteract bias in the MLE include Warm (1989) weighted maximum likelihood estimation (WLE). Although similar to the robust MLE in that the likelihood function is adjusted, WLE addresses bias inherent in the MLE (e.g., due to short test length) rather than bias due to aberrant responding. To address bias in the latent trait due to aberrant responding, one proposed solution is to weigh item responses linearly based on their position on a test. Items appearing later on an assessment are weighed less in ability estimation than the earlier items when performance decline is of concern, and vice versa when performance increase is of concern (Wise et al., 2023). However, this method solely pertains to strict performance decline/incline, and cannot be generalized to a wider range of response disturbances. The weights are also arbitrarily determined by item position, instead of being data-driven.

More general and systematic robust ML estimators for unidimensional IRT (UIRT) and multidimensional IRT (MIRT) models with dichotomous data have been developed for estimating the latent trait (Filonczuk et al., 2022; Mislevy & Bock, 1982; Schuster & Yuan, 2011). These robust estimators are designed to mitigate the effects of response disturbances, on the basis that outlying cases indicate more aberrant behavior. Item responses that poorly fit the IRT model are downweighted such that they contribute less to the estimation of the respondent’s ability. The estimators are able to target a variety of response disturbances and are not restricted to specific aberrant behavior. Both Huber and bisquare weight functions (Huber, 1981; Mosteller & Tukey, 1977) are utilized, which are described later in more detail.

However, these robust IRT developments have been limited to dichotomous data. No robust estimator has been developed for estimating latent traits from polytomously scored Likert-scale items. Given the wide application of Likert-scale items in psychological and educational assessments, we propose a robust estimator using the GRM.

## Methods

According to the GRM, the probability that a subject *i* responds in or above a category *k* for item *j* is

1 $$\begin{aligned} P^*_{jk}(\theta _i) = P(X_{ij} \ge k| \theta _i) = \frac{1}{1+ e^{-Da_j (\theta _i-b_{jk})}}, \end{aligned}$$

Embretson and Reise (2000), where $a_j$ is the item discrimination parameter applied to all category boundary functions for item *j*. *D* is a constant fixed to 1.7, commonly applied in order to scale the item parameters to that of a normal ogive model. Suppose there are *K* categories and $K-1$ threshold parameters ($b_{j,1}; ... b_{j,K-1}$), where the location parameter $b_{jk}$ separates response category *k* and $k+1$ ($k=1,..K-1$). The probability of endorsing exactly category *k* is therefore:

2 $$\begin{aligned} P_{jk}(\theta ) = P^*_{j,k}(\theta ) - P^*_{j,k+1}(\theta ), \end{aligned}$$

where $$P^*_{j1}(\theta ) \equiv 1.0$$ and $$P^*_{jK}(\theta ) \equiv 0.0.$$

The probability that a response falls in a category *k* for an individual with latent trait $\theta _i$ is

$$\begin{aligned} P(X_{ij}=k|\theta _i) = P_{jk}(\theta _i) = \prod ^K_{k=1} P_{jk}(\theta _i)^{u_{ijk}}, \end{aligned}$$

where $u_{ijk}$ is an indicator variable:

3 $$\begin{aligned} u_{ijk} = {\left\{ \begin{array}{ll} 1 & \text {if } X_{ij} = k; \\ 0 & \text {otherwise.} \end{array}\right. } \end{aligned}$$

We assume the number of categories remains constant for all *J* items on the assessment. In ML estimation, we estimate the latent trait by first finding the likelihood of $\theta _i$ given the response vector $\varvec{x}_i = (x_{i1}, x_{i2}, ..., x_{iJ}),$

4 $$\begin{aligned} L(\theta _i|\varvec{x}_i) = \prod ^J_{j=1}\prod ^K_{k=1} P_{jk}(\theta _i)^{u_{ijk}}. \end{aligned}$$

The log-likelihood,

5 $$\begin{aligned} \text {log}L(\theta _i|\varvec{x}_i) = \sum ^J_{j=1} \sum ^K_{k=1} u_{ijk}\text {log}P_{jk}(\theta _i), \end{aligned}$$

is maximized by setting its first derivative equal to 0 and solving for $\theta _i$.

In robust ML estimation, the likelihood of the item response function is weighted by a function $w_{ij}$, resulting in the weighted likelihood $WL(\theta _i|\varvec{x}_i)$:

6 $$\begin{aligned} WL(\theta _i|\varvec{x}_i) = \prod ^J_{j=1}\left( \prod ^K_{k=1} P_{jk}(\theta _i)^{u_{ijk}}\right) ^{w_{ij}} \end{aligned}$$

(Hu, 1994). The resulting weighted log-likelihood (Eq. 7) demonstrates how each item has a unique weighted contribution when determining the overall score:

7 $$\begin{aligned} \text {log}WL(\theta _i|\varvec{x}_i) = \sum ^J_{j=1} w_{ij} \sum ^K_{k=1} u_{ijk}\text {log}P_{jk}(\theta _i). \end{aligned}$$

Assigning an item a weight of zero is the equivalent of removing the response from estimation. Setting $w_j$ equal for all *J* items for a subject gives a weighted log-likelihood equivalent to that in ML estimation (Eq. 5) for finding the most likely $\theta$.

The Newton–Raphson method is then used to estimate $\theta$ iteratively with

8 $$\begin{aligned} \hat{\theta }_{i,(t+1)} = \hat{\theta }_{i,(t)}- \frac{\partial \text {log} WL(\hat{\theta }_{i,(t)})/ \partial \theta _i}{\partial ^2 \text {log} WL(\hat{\theta }_{i,(t)})/ \partial \theta _i^2}, \end{aligned}$$

where *t* is the $t^{th}$ iteration. The first and second derivatives of the weighted log-likelihood with respect to $\theta _i$ are found as follows:

9 $$\begin{aligned} \frac{\partial \text {log}WL}{\partial \theta _i}= &  \sum ^J_{j=1} w_{ij}\sum ^K_{k=1} u_{ijk} a_j \nonumber \\ &  \times \left( \frac{P^*_{j,k-1}(\theta _i)Q^*_{j,k-1}(\theta _i) - P^*_{j,k}(\theta _i)Q^*_{j,k}(\theta _i)}{P_{j,k}} \right) ,\nonumber \\ \end{aligned}$$

and

10 $$\begin{aligned} \frac{\partial ^2 \text {log} WL}{\partial \theta _i^2}= &  \sum ^J_{j=1} w_{ij}\sum ^K_{k=1} u_{ijk} a_j^2\nonumber \\ &  \times \left( \frac{P^*_{j,k-1}(\theta _i)Q^*_{j,k-1}(\theta _i)(Q^*_{j,k-1}(\theta _i)-P^*_{j,k-1}(\theta _i))}{P_{jk}(\theta _i)} \right. \nonumber \\ &  \left. -\frac{P^*_{j,k}(\theta _i) Q^*_{j,k}(\theta _i)(Q^*_{j,k}(\theta _i)-P^*_{j,k}(\theta _i))}{P_{jk}(\theta _i)} \right. \\ &  \left. -\frac{(P^*_{j,k-1}(\theta _i) Q^*_{j,k-1}(\theta _i) -P^*_{j,k}(\theta _i)Q^*_{j,k}(\theta _i))^2}{P_{j,k}^2(\theta _i)} \right) .\nonumber \end{aligned}$$

As suggested by Schuster and Yuan (2011), the MLE is used as a starting value for $\hat{\theta }_{i,(t=0)}$ in the robustified Newton–Raphson algorithm. In our simulation and applied analyses, convergence was deemed reached when the log-likelihoods between consecutive iterations of the Newton-Raphson algorithm fell within a difference of 0.01. If a trait estimate converged outside the interval [-3.0, 3.0], the estimate was fixed to the endpoint closer in value (i.e., an estimate less than -3.0 was replaced with -3.0, and an estimate greater than 3.0 was replaced with 3.0), consistent with common practice in IRT research (Patton et al., 2014). Both of these practices were used for both ML and robust estimation in our simulations and applied example.

Standard errors of each estimate were obtained using the Huber-White sandwich estimator, a technique for estimating the variance of the MLE under a misspecified model (Huber, 1967; White, 1980). In the case of aberrant responses, the main IRT model is misspecified for them. Hence, the Huber–White SE is more appropriate than the usual Fisher-information-based SE for the MLE. The standard error (*SE*) of $\hat{\theta }_i$ for subject *i* is estimated as $SE_{\hat{\theta }_i}=\sqrt{\hat{V}_i}$ where

$$\hat{V}_i=(-A_i)^{-1}B_i(-A_i)^{-1}.$$

$A_i$ is the second derivative of the weighted log-likelihood with respect to $\theta$ (Eq. 10) evaluated at $\hat{\theta }_i:$

11 $$\begin{aligned} A_i=\frac{\partial ^2 \text {log} L}{\partial \theta ^2} \bigg | _{\hat{\theta }_i} . \end{aligned}$$

Using Eq. 9, $B_i$ is estimated as

12 $$\begin{aligned} B_i=\left[ \frac{\partial \text {log}WL}{\partial \theta } \bigg | _{\hat{\theta }_i} \right] ^2. \end{aligned}$$

If the model is correct, $-A$ should equal *B*,  and $\hat{V}$ simplifies to the inverse of $-A$. 95% confidence intervals were calculated with the following expressions

13 $$\begin{aligned} \left[ \hat{\theta }_i- 1.96*SE_{\hat{\theta }_i}, \hat{\theta }_i+ 1.96*SE_{\hat{\theta }_i} \right] . \end{aligned}$$

Coverage rates were then computed as the proportion of subjects whose true $\theta$ lays within the 95% confidence interval, over all replications.

### Weighting mechanisms

It is essential to have a systematic and data-driven way to properly assign weights. Oftentimes the weight is a function of a residual $r_{ij}$, which indicates the “deviation” of an observation from normal or expected behavior. In the IRT literature, $r_{ij}$ is largely determined by the misfit between observed and expected (or model-based) responses, assuming that the IRT model fits well regular response data. The goal of the weighting function is to downweight contributions of items with large residuals. Previous residuals for robust estimation of ability with dichotomous data have drawn on item information to detect misfitting item responses when little information is to be expected from a subject on an item (Filonczuk et al., 2022; Mislevy & Bock, 1982; Schuster & Yuan, 2011). Previous residuals for responses on the GRM have been defined as a function of the difference between the observed response $X_{ij}$ and expected score $E[X_{ij}|\hat{\theta }_i] = \sum _{k=1}^KkP_{j,k}(\hat{\theta }_i)$, such as $r_{ij} = X_{ij} - E[X_{ij}|\hat{\theta }_i]$ used in CUSUM procedures to evaluate person-fit (Van Krimpen-Stoop & Meijer, 2002). Although a variety of residuals could be used, we propose using the standardized residual

14 $$\begin{aligned} r_{ij} = \frac{X_{ij} - E[X_{ij}|\hat{\theta }_i]}{\sigma _{X_{ij}}}. \end{aligned}$$

The residual is standardized by $\sigma _{X_{ij}}$ where

15 $$\begin{aligned} \sigma _{X_{ij}}^2 = \sum _{k=1}^K (X_{ijk}-E[X_{ij}|\hat{\theta }_i])^2P_{jk}(\hat{\theta }_i). \end{aligned}$$

The resulting standardized residual follow asymptotically the standard normal distribution, which allows for easy interpretation.

**Fig. 1:**
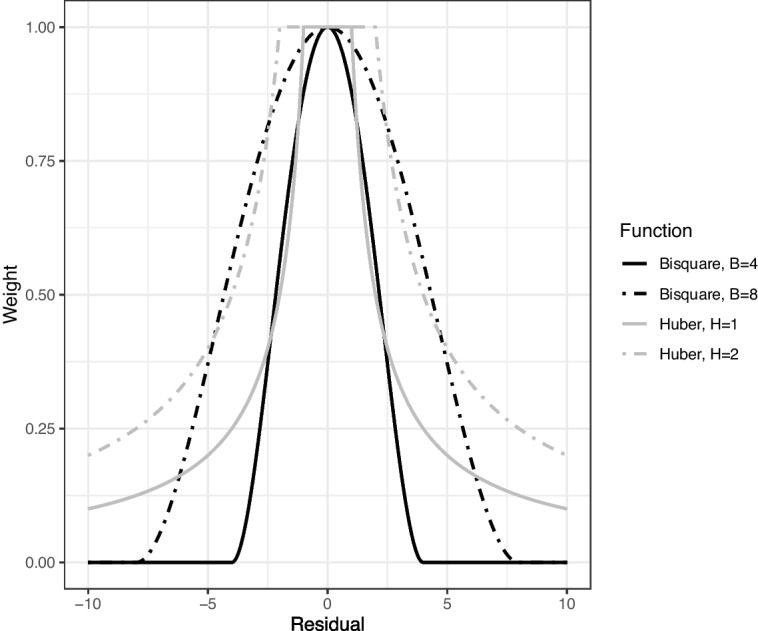
Weights as a function of the residual (*black* = bisquare weights, *grey* = Huber weights). *Solid lines* represent the tuning parameters used in the study (*B*=4.0 and *H*=1.0). *Dashed lines* represent weights when using higher tuning parameters (*B*=8.0 and *H*=2.0)

Given properly defined residuals that characterize how “suspicious” or misfitting a response is, a weight can be assigned to each response. In this study, two commonly used weight functions are considered: bisquare (Mosteller & Tukey, 1977),

16 $$\begin{aligned} w(r_j) = {\left\{ \begin{array}{ll} [1-(r_{ij}/B)^2]^2 & \text { if } |r_{ij}| \le B \\ 0 & \text { if } |r_{ij}| > B \end{array}\right. } \end{aligned}$$

and Huber (Huber, 1981),

17 $$\begin{aligned} w(r_{ij}) = {\left\{ \begin{array}{ll} 1 & \text { if } |r_{ij}| \le H \\ H/|r_{ij}| & \text { if } |r_{ij}| > H, \end{array}\right. } \end{aligned}$$

where *B* and *H* are tuning parameters that regulate the proportion of item responses and the extent to which they are downweighted. Prior studies have incorporated these weight functions when robustly estimating latent traits, so both are considered in our study (Mislevy & Bock, 1982; Schuster & Yuan, 2011). Figure 1 illustrates the weight functions across different residuals with different values for the tuning parameters, *B* and *H*. A greater absolute residual indicates a greater misfit. Therefore, we wish to downweight these cases more. As the residual increases in absolute value, less weight is given, while residuals close to 0.0 are given weights close to 1.0. Tuning parameters *H* and *B* regulate the proportion and degree of the item responses being downweighted. A higher tuning parameter leads to less downweighting. For example, when we use $B = 8.0$ in comparison to $B=4.0$ (the dashed black line in comparison to the solid black line in Fig. 1), item responses with residuals greater in magnitude receive higher weights; thus, such observations are downweighted less. The same effect occurs when we increase the Huber tuning parameter from $H=1.0$ to $H=2.0$, shifting from the grey solid line to the grey dashed line.

To sum up, our robust estimation procedure operates as follows. First, we find the MLE for each respondent. Second, we calculate the residuals on all items for each respondent, identifying the degree of mismatch between the expected and observed item responses. Third, a weighting mechanism is chosen, and a weight is assigned to each item response as a function of the residual. Fourth, the robust estimate for each individual is updated incorporating these weights. Steps two through four are repeated, calculating the residual as a function of the latest updated latent trait estimate, until the Newton–Raphson algorithm converges.

## Simulation study

While this estimation procedure is capable of counteracting a wide variety of response disturbances, our main simulation examines two examples of commonly observed aberrant responses in low-stakes assessments: back random responding (BRR) and overlooking reverse-coded statements. BRR occurs when respondents initially respond to items according to their true latent trait, then switch to a careless response pattern, which is continued for the remaining items. Previous studies have reported that careless responding primarily occurs on the latter items of an assessment, particularly when motivation is low for taking the assessment (Clark et al., 2003). Because of its prevalence, we chose to model BRR as a response disturbance in our simulations.

Furthermore, reverse-coded items are worded in a sentiment opposite from the scale (i.e., negatively worded), but are assumed to measure the same underlying construct (Hughes, 2009). An example item is “I feel unhappy” in a scale that measures happiness. A greater score on a reverse-coded item indicates a lower level of the latent trait measured by the item, and vice versa. They are often included to provoke the subject to pay more attention and process item content individually instead of forming response sets (i.e., responding according to general attitudes towards the test as a whole; Weems et al., 2003). In addition, adding reverse-coded items may help avoid biased responding that can result if all items are worded in a socially desirable direction (Weems et al., 2006). When including these items, it is assumed that subjects will respond to negatively worded items in a manner opposite to the positively worded items. However, subjects and researchers may both violate this assumption when encountering reverse-coded items. First, subjects may be inattentive and overlook reverse-coded items, responding erroneously (Hughes, 2009). Reading and interpreting reverse-coded items may require more mental effort and be more difficult for subjects, resulting in an aberrant response (Marsh, 1986; Williams & Swanson, 2001) as well. Second, researchers who perform secondary data analysis may be unaware of negatively worded items, due to carelessness on their end or unfamiliarity with the data set. Then, these items may not be properly reverse-coded prior to analysis. These aberrant behaviors violate the IRT model, since the response does not reflect the subject’s true latent trait when calculating the score. Thus, we use robust estimation in lieu of ML estimation to provide more accurate latent trait scores.

In our simulation study, we varied the following properties of a test: the number of categories on each Likert-type item, the test length, the severity, or proportion of items that are disturbed in each response vector, and the type of aberrant response: BRR or overlooked reverse coding.

Likert-type scales are commonly developed with either three, five, or seven categories, where each number of categories has its respective advantages (Aybek & Toraman, 2022). Therefore, we simulated tests with three, five, and seven categories, keeping the number of categories constant for all items within each test (e.g., a test could not have some items with three categories and some items with five categories).

Tests were generated with lengths of 10, 30, 50, and 70 items. Although multidimensional psychological inventories can include hundreds of items, such as the 567-item MMPI-2 (Sellbom & Anderson, 2013), we are concerned with unidimensional scales, so in this study the maximum test length was set at 70.

The robust estimation is most successful when the subject exhibits some behavior true to their latent trait and not all responses are aberrant, otherwise complete data removal would be sufficient. Previous studies have set the severity, or proportion of aberrant items, between 10%-50% (Meade & Craig, 2012). Therefore, we examined two severity conditions: 10% and 30%.

In effect, under back random responding, random guessing began after 90% or 70% (severities of 0.10 and 0.30, respectively) of the test. At this change point, each response category had an equal probability (e.g., 1/*K*) of being selected according to the discrete uniform distribution *U*[1, *K*], for an item with *K* categories.

In the neglected reverse coded simulations (i.e., subjects are inattentive to the negatively worded items), 10% or 30% of randomly selected items from each test were designated as negatively worded. Aberrant responses to these items were generated as follows. First, the item response $u_j$ was generated based on the subject’s probabilities of endorsing each category according to their true ability. The response was then disturbed by being reversed in value, such that

$$\begin{aligned} u^*_{ij} = {\left\{ \begin{array}{ll} u_{ij} & \text {if subject}\, i \, \text {is attentive to item } j \\ K+1- u_{ij} & \text {if subject}\, i \, \text {is inattentive to item } j \end{array}\right. } \end{aligned}$$

where $u^*_{ij}$ is the new response to replace $u_{ij}$. For example, on a 5-pt Likert scale ranging from 1 to 5, consider a subject with a high latent trait who is likely to respond 1 to the negatively worded item. After reverse coding, the response will be coded as 5, reflecting the subject’s true latent trait. However, if the subject fails to recognize it’s a negatively worded item, their response would be 5. After reverse coding, the response would be 1, which indicates a low latent trait and not their true latent trait.

In total, we generated a $3\times 4\times 2\times 2$ simulation design, incorporating three levels of item response categories, four test lengths, two severity levels, and two types of aberrant responses. For item parameter generation, we follow Dodd et al. (1989), who suggest generating $b_j$s at random uniformly across the latent trait continuum, while varying the characteristics of the category threshold parameters within each item. In our study, we simulated $b_j \sim Unif(-2.5, 2.5)$ to generate $K-1$ values, one value for each threshold parameter, for each item *j*. Within each item, these values were sorted in ascending order such that the highest value corresponded with the greatest threshold. Item discrimination parameters $a_i$s have been previously simulated according to *Unif*(0.90, 2.15) (Dodd et al., 1989). Therefore, our items used values from [0.90, 2.15] with equal increments between each value; e.g., for a 10-item assessment, the 10 $a_i$s would be (0.900, 0.943, 0.986, ..., 2.106, 2.150). All item parameters were treated as fixed across all replications for each of the 24 conditions.

True $\theta$s from [-2.0, 2.0] in increments of 0.25 were used to generate data from each of the tests. Six hundred replications of each $\theta$ were used, forming 600 response vectors given the item parameters based on the following steps. Probabilities of responding in each category were generated for each of these true $\theta$s according to the item parameters and the item response function (Eq. 2). Polytomous data was generated through sampling, using these probabilities as the probability of landing in each category.

Three trait estimates were obtained: the MLE, the bisquare-weighted robust estimate, and the Huber-weighted robust estimate. The same response vector was used for all three estimators. Initial values of zero were used in the Newton–Raphson algorithm to estimate the MLE. Following past practices in robust IRT literature, the MLE was then used as the initial value to estimate the robust estimate, using zero in cases where the MLE did not converge. The average bias and MSE were evaluated for the three latent trait estimates. If the estimate did not converge within 30 iterations or approached infinity, the estimate was considered missing and removed from our calculation of bias and MSE. If more than 30% of the estimates for a trait level on a certain test design did not converge, we did not report the bias and MSE for the trait level on that test design.

**Table 1:** Bias using maximum likelihood (ML), biweight (BI, *B = 4*), and Huber (HU, *H = 1*) estimation of the latent trait when there is back random responding in the data at a severity of 10%. (Nonconvergence rates higher than 30% displayed in parentheses)

			$$\theta $$								
	*K*	*J*	−2	−1.5	−1	−0.5	0	0.5	1	1.5	2
ML	3	10	(79.5%)	(43.3%)	0.18	0.08	−0.05	−0.18	−0.28	(67.5%)	(89.5%)
		30	0.42	0.37	0.28	0.17	0.06	−0.01	−0.09	−0.15	−0.21
		50	0.23	0.12	0.02	−0.04	−0.08	−0.14	−0.24	−0.30	−0.31
		70	0.31	0.23	0.15	0.07	−0.01	−0.08	−0.16	−0.19	−0.25
	5	10	(66.8%)	0.15	−0.03	−0.17	−0.27	−0.30	−0.24	(35.5%)	(80.8%)
		30	(33.7%)	0.18	0.12	0.03	−0.02	−0.07	−0.10	−0.17	−0.22
		50	(48.8%)	0.16	0.10	0.05	0.00	−0.05	−0.11	−0.17	−0.31
		70	0.24	0.17	0.13	0.09	0.04	−0.02	−0.09	−0.15	(33.2%)
	7	10	(78.7%)	0.29	0.20	0.17	0.11	0.01	−0.06	−0.20	(44.0%)
		30	(89.8%)	0.15	0.05	0.02	−0.01	−0.06	−0.09	−0.15	(70.3%)
		50	(80.3%)	0.11	0.07	0.04	−0.01	−0.04	−0.09	−0.15	(84.0%)
		70	(91.0%)	0.14	0.10	0.07	0.03	−0.02	−0.06	−0.13	(88.0%)
HU	3	10	−0.08	−0.05	0.13	0.10	−0.03	−0.12	−0.08	0.04	0.05
		30	−0.10	0.02	0.07	0.05	0.02	−0.01	−0.02	−0.03	0.07
		50	−0.08	−0.04	−0.08	−0.02	0.01	0.02	−0.07	−0.11	0.06
		70	−0.01	0.01	−0.02	0.01	0.04	0.01	−0.02	−0.04	0.02
	5	10	0.01	−0.04	−0.05	−0.10	−0.13	−0.12	−0.04	0.09	0.21
		30	−0.06	0.04	0.03	−0.04	−0.02	−0.03	0.02	0.05	0.07
		50	−0.04	0.03	0.02	0.01	0.03	0.00	−0.03	−0.01	0.02
		70	0.00	0.03	0.01	0.03	0.05	0.00	−0.04	−0.04	0.02
	7	10	−0.15	0.02	0.09	0.10	0.09	0.02	−0.04	−0.08	−0.06
		30	−0.04	0.01	0.03	0.02	0.00	−0.03	−0.03	−0.01	0.02
		50	−0.02	0.01	0.03	0.01	−0.01	−0.03	−0.02	−0.03	0.00
		70	−0.04	0.04	0.05	0.02	0.02	−0.01	−0.03	−0.04	−0.02
BI	3	10	−0.33	−0.12	0.12	0.09	−0.02	−0.12	−0.08	0.09	0.11
		30	−0.13	−0.03	0.00	0.02	0.00	−0.03	0.00	0.00	0.09
		50	−0.13	−0.09	−0.10	−0.02	0.03	0.05	−0.04	−0.06	0.07
		70	−0.05	−0.04	−0.06	0.01	0.03	0.02	0.01	0.00	0.05
	5	10	−0.09	−0.05	−0.04	−0.08	−0.10	−0.09	−0.02	0.07	(35.0%)
		30	−0.11	0.00	0.00	−0.05	−0.03	0.00	0.04	0.07	0.08
		50	−0.07	−0.01	0.00	0.00	0.02	0.01	−0.01	0.02	0.08
		70	−0.04	−0.01	−0.02	0.02	0.03	0.01	−0.02	−0.01	0.08
	7	10	−0.29	−0.06	0.06	0.08	0.07	0.01	−0.03	−0.05	−0.03
		30	−0.08	−0.01	0.02	0.01	0.00	−0.01	−0.01	0.02	0.05
		50	−0.05	−0.01	0.01	0.01	−0.02	−0.01	0.00	−0.01	0.03
		70	−0.07	0.00	0.02	0.01	0.01	0.00	−0.02	−0.01	0.01

**Table 2:** Bias using maximum likelihood (ML), biweight (BI, *B = 4*), and Huber (HU, *H = 1*) estimation of the latent trait when there is back random responding in the data at a severity of 30%. (Nonconvergence rates higher than 30% displayed in parentheses)

			$$\theta $$								
	*K*	*J*	−2	−1.5	−1	−0.5	0	0.5	1	1.5	2
ML	3	10	(58.2%)	(30.2%)	0.62	0.50	0.29	0.01	−0.26	(48.0%)	(65.7%)
		30	0.95	0.71	0.44	0.22	0.01	−0.19	−0.36	−0.55	−0.71
		50	0.82	0.57	0.35	0.16	−0.01	−0.21	−0.41	−0.58	−0.74
		70	0.71	0.50	0.30	0.12	−0.08	−0.29	−0.49	−0.66	−0.78
	5	10	(38.3%)	0.35	0.07	−0.13	−0.25	−0.37	−0.47	−0.70	(33.8%)
		30	0.72	0.53	0.33	0.13	−0.07	−0.23	−0.41	−0.55	−0.74
		50	0.63	0.45	0.26	0.10	−0.05	−0.18	−0.34	−0.52	−0.75
		70	0.62	0.48	0.34	0.24	0.11	−0.06	−0.21	−0.36	−0.58
	7	10	0.94	0.70	0.56	0.43	0.31	0.15	0.00	−0.30	−0.63
		30	0.60	0.37	0.21	0.10	−0.05	−0.19	−0.36	−0.50	−0.65
		50	0.58	0.44	0.31	0.17	0.02	−0.12	−0.26	−0.43	−0.60
		70	0.59	0.44	0.32	0.18	0.02	−0.12	−0.25	−0.39	−0.57
HU	3	10	−0.08	−0.07	0.16	0.25	0.19	0.14	0.11	−0.09	−0.36
		30	0.01	0.09	0.18	0.13	−0.01	−0.10	−0.12	−0.14	−0.07
		50	0.12	0.09	0.10	0.05	0.03	−0.03	−0.14	−0.19	−0.11
		70	0.06	0.07	0.04	0.07	0.01	−0.09	−0.15	−0.15	−0.09
	5	10	0.12	−0.01	−0.11	−0.14	−0.15	−0.12	−0.04	−0.13	−0.20
		30	0.20	0.25	0.18	0.05	−0.08	−0.16	−0.15	−0.05	−0.03
		50	0.13	0.14	0.08	0.01	−0.02	−0.04	−0.10	−0.16	−0.19
		70	0.09	0.11	0.09	0.09	0.09	0.00	−0.07	−0.11	−0.18
	7	10	0.08	0.13	0.20	0.19	0.20	0.15	0.05	−0.10	−0.28
		30	0.14	0.12	0.07	0.05	−0.01	−0.08	−0.15	−0.17	−0.13
		50	0.14	0.16	0.13	0.09	0.00	−0.05	−0.08	−0.16	−0.18
		70	0.15	0.16	0.14	0.06	−0.01	−0.06	−0.08	−0.14	−0.17
BI	3	10	−0.41	−0.18	0.14	0.25	0.17	0.15	0.15	−0.02	−0.26
		30	−0.14	−0.12	0.04	0.08	−0.03	−0.10	−0.05	−0.02	0.00
		50	−0.05	−0.05	0.01	0.00	0.03	0.01	−0.07	−0.06	0.01
		70	−0.08	−0.07	−0.05	0.04	0.02	−0.06	−0.04	−0.01	0.03
	5	10	0.00	−0.06	−0.15	−0.15	−0.13	−0.08	0.02	−0.09	−0.19
		30	0.04	0.11	0.11	0.03	−0.07	−0.11	−0.07	0.06	0.07
		50	0.00	0.01	0.01	0.00	−0.02	0.00	−0.03	−0.06	−0.04
		70	−0.04	−0.02	0.02	0.05	0.06	0.02	−0.02	−0.01	−0.02
	7	10	−0.11	−0.02	0.08	0.11	0.16	0.14	0.04	−0.07	−0.23
		30	0.03	0.03	0.02	0.02	0.00	−0.02	−0.06	−0.06	−0.01
		50	0.03	0.06	0.05	0.05	−0.01	−0.01	−0.01	−0.06	−0.05
		70	0.02	0.04	0.06	0.03	−0.02	−0.02	−0.03	−0.05	−0.05

**Table 3:** Sandwich standard error using maximum likelihood (ML), biweight (BI, *B = 4*), and Huber (HU, *H = 1*) estimation of the latent trait when there is back random responding in the data at a severity of 10%. (Nonconvergence rates higher than 30% displayed in parentheses)

			$$\theta $$								
	*K*	*J*	−2	−1.5	−1	−0.5	0	0.5	1	1.5	2
ML	3	10	(79.5%)	(43.3%)	0.71	0.76	0.77	0.72	0.59	(67.5%)	(89.5%)
		30	0.62	0.47	0.32	0.21	0.21	0.28	0.34	0.34	0.36
		50	0.39	0.30	0.22	0.17	0.18	0.23	0.26	0.29	0.32
		70	0.28	0.23	0.18	0.14	0.14	0.19	0.25	0.28	0.29
	5	10	(66.8%)	0.47	0.42	0.40	0.42	0.46	0.46	(35.5%)	(80.8%)
		30	(33.7%)	0.31	0.23	0.17	0.17	0.21	0.28	0.38	0.53
		50	(48.8%)	0.24	0.17	0.13	0.15	0.18	0.20	0.25	0.34
		70	0.27	0.23	0.18	0.13	0.12	0.13	0.14	0.18	(33.2%)
	7	10	(78.7%)	0.59	0.44	0.34	0.33	0.36	0.40	0.46	(44.0%)
		30	(89.8%)	0.25	0.20	0.17	0.17	0.19	0.23	0.30	(70.3%)
		50	(80.3%)	0.22	0.19	0.17	0.15	0.15	0.17	0.21	(84.0%)
		70	(91.0%)	0.19	0.15	0.12	0.11	0.12	0.14	0.18	(88.0%)
HU	3	10	1.11	0.74	0.79	0.95	1.01	0.86	0.60	0.58	1.14
		30	0.96	0.57	0.39	0.25	0.22	0.30	0.35	0.36	0.50
		50	0.62	0.35	0.25	0.18	0.19	0.25	0.28	0.31	0.39
		70	0.35	0.26	0.20	0.15	0.15	0.21	0.27	0.29	0.34
	5	10	0.74	0.53	0.46	0.42	0.40	0.37	0.40	0.76	2.51
		30	0.64	0.35	0.25	0.18	0.17	0.21	0.30	0.45	0.69
		50	0.39	0.26	0.18	0.14	0.16	0.19	0.21	0.29	0.51
		70	0.31	0.24	0.18	0.13	0.13	0.13	0.15	0.20	0.32
	7	10	1.65	0.67	0.44	0.35	0.35	0.38	0.43	0.54	0.75
		30	0.46	0.28	0.21	0.18	0.17	0.19	0.24	0.35	0.53
		50	0.37	0.24	0.20	0.18	0.16	0.15	0.17	0.22	0.34
		70	0.33	0.20	0.16	0.13	0.12	0.13	0.15	0.20	0.29
BI	3	10	2.66	1.26	0.84	0.95	1.00	0.87	0.61	0.65	1.29
		30	1.08	0.61	0.42	0.26	0.22	0.30	0.36	0.37	0.53
		50	0.75	0.37	0.26	0.18	0.20	0.25	0.29	0.31	0.39
		70	0.38	0.27	0.21	0.15	0.15	0.22	0.28	0.30	0.37
	5	10	1.19	0.61	0.47	0.42	0.40	0.37	0.44	0.76	(35.0%)
		30	0.72	0.37	0.26	0.18	0.17	0.22	0.31	0.47	0.70
		50	0.42	0.26	0.19	0.14	0.16	0.19	0.21	0.31	0.57
		70	0.33	0.25	0.19	0.13	0.13	0.14	0.16	0.21	0.35
	7	10	2.49	0.89	0.47	0.35	0.35	0.38	0.44	0.56	0.76
		30	0.55	0.29	0.22	0.18	0.18	0.20	0.25	0.36	0.56
		50	0.39	0.24	0.20	0.18	0.16	0.15	0.17	0.23	0.35
		70	0.35	0.21	0.16	0.13	0.12	0.13	0.15	0.20	0.32

**Table 4:** Sandwich standard error using maximum likelihood (ML), biweight (BI, *B = 4*), and Huber (HU, *H = 1*) estimation of the latent trait when there is back random responding in the data at a severity of 30%. (Nonconvergence rates higher than 30% displayed in parentheses)

			$$\theta $$								
	*K*	*J*	−2	−1.5	−1	−0.5	0	0.5	1	1.5	2
ML	3	10	(58.2%)	(30.2%)	0.73	0.72	0.69	0.64	0.57	(48.0%)	(65.7%)
		30	0.44	0.35	0.27	0.22	0.21	0.25	0.30	0.33	0.34
		50	0.26	0.21	0.18	0.17	0.19	0.22	0.25	0.27	0.29
		70	0.23	0.20	0.17	0.14	0.14	0.16	0.20	0.24	0.27
	5	10	(38.3%)	0.45	0.43	0.42	0.43	0.45	0.46	0.46	(33.8%)
		30	0.30	0.25	0.20	0.17	0.17	0.20	0.24	0.29	0.36
		50	0.24	0.19	0.15	0.13	0.15	0.18	0.19	0.21	0.23
		70	0.23	0.19	0.15	0.13	0.12	0.13	0.14	0.16	0.19
	7	10	0.54	0.45	0.38	0.35	0.35	0.38	0.41	0.45	0.50
		30	0.26	0.22	0.19	0.17	0.17	0.18	0.20	0.24	0.31
		50	0.22	0.20	0.18	0.16	0.15	0.15	0.16	0.18	0.21
		70	0.19	0.17	0.14	0.12	0.11	0.12	0.13	0.15	0.18
HU	3	10	1.06	0.75	0.67	0.77	0.80	0.70	0.54	0.49	0.60
		30	0.91	0.56	0.36	0.24	0.22	0.27	0.33	0.35	0.47
		50	0.46	0.30	0.20	0.17	0.20	0.24	0.27	0.30	0.36
		70	0.34	0.24	0.19	0.15	0.15	0.19	0.25	0.28	0.32
	5	10	0.77	0.57	0.48	0.44	0.42	0.40	0.43	0.61	1.33
		30	0.46	0.30	0.23	0.18	0.17	0.19	0.25	0.38	0.63
		50	0.32	0.23	0.17	0.13	0.15	0.18	0.20	0.25	0.38
		70	0.29	0.23	0.17	0.12	0.13	0.14	0.15	0.20	0.29
	7	10	1.14	0.60	0.39	0.33	0.35	0.40	0.47	0.58	0.73
		30	0.40	0.27	0.21	0.17	0.17	0.19	0.22	0.29	0.45
		50	0.30	0.21	0.19	0.17	0.16	0.16	0.17	0.21	0.30
		70	0.27	0.19	0.15	0.13	0.12	0.12	0.14	0.18	0.25
BI	3	10	2.74	1.31	0.78	0.76	0.81	0.72	0.56	0.56	0.79
		30	1.22	0.71	0.43	0.25	0.22	0.28	0.34	0.37	0.50
		50	0.62	0.34	0.23	0.18	0.20	0.25	0.28	0.31	0.39
		70	0.43	0.28	0.21	0.15	0.15	0.20	0.26	0.29	0.35
	5	10	1.28	0.65	0.51	0.46	0.43	0.41	0.47	0.65	1.27
		30	0.59	0.33	0.24	0.18	0.17	0.20	0.26	0.44	0.71
		50	0.39	0.25	0.18	0.14	0.15	0.18	0.21	0.28	0.47
		70	0.34	0.25	0.18	0.13	0.13	0.14	0.16	0.22	0.37
	7	10	1.72	0.85	0.45	0.34	0.35	0.40	0.48	0.61	0.80
		30	0.49	0.29	0.22	0.18	0.18	0.20	0.24	0.33	0.53
		50	0.35	0.23	0.20	0.18	0.16	0.16	0.18	0.23	0.35
		70	0.32	0.21	0.16	0.13	0.12	0.13	0.15	0.19	0.29

Tuning parameters $B=4.0$ and $H=1.0$ were used for the bisquare and Huber weight functions, respectively, in all simulations. These values were chosen through a series of pilot simulations in which *B* and *H* were each varied, and the optimal result was the value that produced the most reduced bias and MSE across the test conditions in our main simulations. For brevity, we omit these results from the paper but can provide them upon request. Although we used a limited number of conditions to come up with these values, the parameter values $B=4.0$ and $H=1.0$ are also consistent with the tuning parameters suggested in previous robust estimation of latent traits (Mislevy & Bock, 1982; Schuster & Yuan, 2011). However, for data with low degrees of aberrant responses (e.g., a severity of 10%) generated from tests with few categories (e.g., $K=3$), greater tuning parameters, such as $H=2.0$ and $B=8.0$, tend to optimize the bias and MSE. Using $H=1.0$ and $B=4.0$ in this situation results in a minor overcorrection of the data (i.e., for a MLE with a negative bias, the robust estimate may produce positive bias). Since increasing the tuning parameter decreases the amount of downweighting applied to the data, these results appear consistent: with fewer response disturbances, less downweighting should be applied to the data.

## Results

Similar patterns were observed for both back random responding and neglected reverse coding, so our findings are generalized to both types of aberrant responding. Due to a large number of simulation conditions, tables from the simulations with neglected reverse coding were omitted from this paper but are available on OSF (https://osf.io/jku4d/).

When an estimation method led to 30% or greater nonconvergence for a certain true trait and test design, the bias and average standard error were not reported. Instead, the nonconvergence rate is reported in parentheses with the results (Tables 1, 2, 3 and 4). This incident occurred predominantly in ML estimation for extreme traits (e.g., $\theta =\pm 1.5$ or even more extreme). However, the robust procedures both tend to result in much lower nonconvergence rates, suggesting that, when the ML estimation fails in convergence, robust estimation may be able to provide a converging estimate.

Overall, the bisquare- and Huber-weighted robust estimates resulted in less bias compared to the MLE. Tables 3 and 4 display the average bias for each of the three estimates (ML, Huber-weighted, and bisquare-weighted estimation) over each test design. For conciseness, a subset of true latent traits from the full simulation is shown ($\theta =$ -2.0, -1.5, -1.0, -0.5, 0.0, 0.5, 1.0, 1.5, and 2.0). Although both estimators were typically effective in reducing the bias, the bisquare weight function tended to produce the smaller bias of the two robust methods. For example, when the severity is 0.10, $J=70$, $K=3$, and $\theta =1.5$, $\hat{\theta }_{ML}$ gives a bias of $-0.19$, while $\hat{\theta }_{H}$ and $\hat{\theta }_{B}$ give $-0.04$ and 0.00, respectively. While the bisquare procedure does not always eliminate the bias, it typically is closer to zero than the Huber-weighted estimate. When *K* increases, the MLE tends to produce smaller bias, but not to the extent of elimination. Likewise, the Huber-and bisquare-weighted estimates provide smaller bias as *K* increases, to the point where the bisquare-weighted estimate almost reaches a negligible bias when $K=7$.

Furthermore, when the severity is lower (Table 1), the bias is less in the MLE compared to instances of greater severity (Table 2). This pattern aligns with our expectations that, as more aberrant responses are present, the estimates will produce more bias. As the severity, number of categories, and test length all decrease, a slight overcorrection tends to appear in the bias by the robust estimators. For example, when $\theta =-2.00$, severity is 0.10, $K=3$, and $J=30$, the bias for $\hat{\theta }_{MLE}$ is positive, 0.40, while that of $\hat{\theta }_{H}$ and $\hat{\theta }_{B}$ is -0.13 and -0.16, respectively. Beyond $J=10$ (with greater *K* and severity) the test length does not have much of an impact on the bias.

Therefore, the robust estimation works best with assessments with more than ten items and/or more than three categories. For such assessments with fewer items and categories, we recommend using robust estimation with caution, though the overcorrection that occurs in shorter tests can be mitigated with a larger tuning parameter.

Average sandwich standard errors for the latent trait estimates can be found in Tables 3 and 4. Standard errors are slightly larger for the robust estimates compared to MLE in some cases of extreme latent traits (e.g., $\pm 2.0$) or cases of short test lengths (e.g., $J=10$), but is otherwise negligible. Previous studies have reported increases in standard error consistent with these results when using robust estimation in general (Carroll & Pederson, 1993) and more precisely in item response models (Schuster & Yuan, 2011). When evaluating estimation procedures, the bias-variance trade-off must be considered: reducing the bias risks an increase in variance. However, the standard error does not increase substantially with the robust estimation in our study, compared to that of the MLE. Therefore, we achieve a desirable bias-variance balance, and the total error is not inflated with robust estimation.

Our findings are consistent with the robust literature. Overall, the robust estimates resulted in less bias along with comparable standard errors, compared to the MLEs. We find that the bisquare-weighted estimate performs better than the Huber-weighted estimate in most cases. This finding contradicts the findings of Schuster and Yuan (2011), which suggested the Huber-weighted estimate outperforms the bisquare-weighted estimate due to a high incidence of infinite estimates with the bisquare weight. Because our study pertains to Likert-type data, as opposed to dichotomous data studied by Schuster and Yuan (2011), less nonconvergence occurs since it is less likely that all responses fall in the lowest/highest response category (similar to all zeros or all 1’s in dichotomous data). Therefore, the issue of infinite estimates does not impact our bisquare estimation here in polytomous items as much as in dichotomous items, and the bisquare weight indeed tends to outperform the Huber weight in the robust estimation.

The Huber weight outperforms the bisquare weight in very few instances, such as when there is both low severity and smaller *K*. Here, the bisquare weight function results in a greater degree of overcorrection than the Huber weight function. Moreover, the Huber weight tends to result in a smaller standard error in short test lengths with more extreme traits. In all other cases, the bisquare weight tends to result in less bias and standard errors comparable to the Huber weight function.

**Table 5:** Coverage rates for the sandwich standard error using maximum likelihood (ML), biweight (BI, *B = 4*), and Huber (HU, *H = 1*) estimation of the latent trait when there is back random responding in the data at a severity of 10%. (Nonconvergence rates higher than 30% displayed in parentheses)

			$$\theta $$								
	*K*	*J*	−2	−1.5	−1	−0.5	0	0.5	1	1.5	2
ML	3	10	(79.5%)	(43.3%)	0.97	0.95	0.91	0.92	0.98	(67.5%)	(89.5%)
		30	0.95	0.89	0.84	0.84	0.99	0.94	0.95	0.98	0.98
		50	0.97	0.96	0.97	0.98	0.91	0.89	0.88	0.88	0.92
		70	0.86	0.87	0.87	0.94	0.96	0.92	0.90	0.95	0.96
	5	10	(66.8%)	0.97	1.00	0.99	0.92	0.92	0.96	(35.5%)	(80.8%)
		30	(33.7%)	0.95	0.92	0.95	0.92	0.94	0.96	0.96	0.98
		50	(48.8%)	0.95	0.90	0.94	0.95	0.97	0.97	0.97	0.95
		70	0.98	0.97	0.90	0.87	0.91	0.93	0.90	0.92	(33.2%)
	7	10	(78.7%)	0.98	0.95	0.94	0.98	0.97	0.97	0.96	(44.0%)
		30	(89.8%)	0.96	0.93	0.92	0.92	0.93	0.94	0.97	(70.3%)
		50	(80.3%)	0.99	0.98	0.96	0.98	0.97	0.95	0.95	(84.0%)
		70	(91.0%)	0.96	0.91	0.88	0.93	0.94	0.95	0.96	(88.0%)
HU	3	10	1.00	1.00	1.00	0.99	0.98	0.97	1.00	1.00	0.98
		30	1.00	0.99	0.96	0.91	0.97	0.95	0.97	1.00	1.00
		50	1.00	1.00	0.99	0.96	0.96	0.98	0.99	0.98	1.00
		70	1.00	0.99	0.97	0.94	0.96	0.99	1.00	1.00	1.00
	5	10	0.98	0.99	0.99	0.98	0.93	0.94	0.94	0.98	0.99
		30	1.00	0.99	0.98	0.97	0.92	0.96	0.98	0.99	1.00
		50	1.00	1.00	0.98	0.95	0.97	0.98	0.99	0.99	1.00
		70	1.00	1.00	0.99	0.94	0.93	0.95	0.96	0.97	1.00
	7	10	1.00	0.98	0.96	0.95	0.96	0.97	0.98	0.98	0.99
		30	1.00	0.99	0.96	0.96	0.97	0.97	0.98	0.99	1.00
		50	1.00	1.00	0.99	0.98	0.98	0.97	0.98	0.99	1.00
		70	1.00	0.99	0.98	0.96	0.95	0.97	0.98	0.99	1.00
BI	3	10	1.00	1.00	1.00	1.00	0.99	0.96	1.00	1.00	0.98
		30	1.00	0.99	0.97	0.94	0.97	0.94	0.96	1.00	1.00
		50	1.00	1.00	0.99	0.95	0.96	0.98	1.00	0.99	1.00
		70	1.00	1.00	0.98	0.94	0.96	0.98	1.00	1.00	1.00
	5	10	0.98	0.98	0.99	0.97	0.92	0.94	0.95	0.99	(35.0%)
		30	1.00	0.99	0.98	0.96	0.90	0.96	0.98	0.99	1.00
		50	1.00	1.00	0.97	0.96	0.97	0.98	1.00	0.99	1.00
		70	1.00	1.00	0.98	0.95	0.92	0.94	0.97	0.99	1.00
	7	10	1.00	0.99	0.96	0.96	0.96	0.96	0.98	0.97	0.99
		30	1.00	0.99	0.98	0.95	0.96	0.96	0.99	0.99	1.00
		50	1.00	1.00	0.99	0.99	0.97	0.96	0.98	0.99	1.00
		70	1.00	1.00	0.98	0.96	0.95	0.97	0.99	0.99	1.00

**Table 6:** Coverage rates for the sandwich standard error using maximum likelihood (ML), biweight (BI, *B = 4*), and Huber (HU, *H = 1*) estimation of the latent trait when there is back random responding in the data at a severity of 30%. (Nonconvergence rates higher than 30% displayed in parentheses)

			$$\theta $$								
	*K*	*J*	−2	−1.5	−1	−0.5	0	0.5	1	1.5	2
ML	3	10	(58.2%)	(30.2%)	0.84	0.76	0.77	0.85	0.97	(48.0%)	(65.7%)
		30	0.43	0.42	0.50	0.71	0.94	0.75	0.68	0.63	0.49
		50	0.16	0.28	0.49	0.78	0.88	0.77	0.62	0.45	0.29
		70	0.17	0.31	0.54	0.77	0.85	0.53	0.34	0.29	0.20
	5	10	(38.3%)	0.79	0.95	0.91	0.80	0.76	0.80	0.75	(33.8%)
		30	0.37	0.44	0.54	0.78	0.84	0.68	0.58	0.49	0.43
		50	0.26	0.39	0.55	0.80	0.80	0.75	0.58	0.30	0.09
		70	0.22	0.34	0.41	0.50	0.68	0.82	0.60	0.39	0.17
	7	10	0.63	0.60	0.57	0.69	0.80	0.91	0.95	0.87	0.72
		30	0.36	0.59	0.71	0.72	0.78	0.69	0.54	0.45	0.44
		50	0.24	0.43	0.55	0.73	0.85	0.78	0.59	0.37	0.19
		70	0.13	0.30	0.39	0.60	0.79	0.72	0.53	0.32	0.13
HU	3	10	0.98	0.98	0.95	0.90	0.84	0.76	0.98	1.00	0.87
		30	0.98	0.85	0.79	0.80	0.92	0.82	0.87	0.95	0.94
		50	0.95	0.88	0.82	0.83	0.88	0.91	0.90	0.88	0.93
		70	0.95	0.92	0.87	0.80	0.87	0.87	0.85	0.94	0.95
	5	10	0.94	0.96	0.96	0.91	0.83	0.87	0.93	0.94	0.94
		30	0.89	0.79	0.78	0.87	0.83	0.73	0.80	0.94	0.98
		50	0.95	0.90	0.88	0.86	0.80	0.87	0.88	0.90	0.90
		70	0.98	0.94	0.88	0.80	0.77	0.90	0.87	0.91	0.91
	7	10	0.95	0.91	0.82	0.86	0.82	0.89	0.97	0.95	0.89
		30	0.95	0.89	0.87	0.82	0.84	0.85	0.82	0.89	0.96
		50	0.93	0.87	0.85	0.87	0.92	0.86	0.88	0.86	0.92
		70	0.96	0.86	0.79	0.85	0.83	0.81	0.86	0.88	0.92
BI	3	10	0.99	0.98	0.94	0.88	0.84	0.78	1.00	1.00	0.92
		30	0.99	0.93	0.83	0.85	0.91	0.79	0.85	0.97	0.97
		50	0.98	0.94	0.87	0.85	0.85	0.90	0.93	0.93	0.98
		70	0.99	0.98	0.89	0.79	0.87	0.87	0.93	0.98	0.99
	5	10	0.95	0.95	0.94	0.89	0.83	0.88	0.96	0.94	0.94
		30	0.95	0.88	0.80	0.88	0.80	0.77	0.86	0.97	0.99
		50	0.98	0.96	0.91	0.85	0.78	0.85	0.93	0.95	0.97
		70	1.00	0.98	0.93	0.83	0.78	0.88	0.91	0.95	0.97
	7	10	0.98	0.95	0.88	0.88	0.83	0.87	0.95	0.94	0.89
		30	0.99	0.93	0.88	0.81	0.80	0.85	0.91	0.95	0.98
		50	0.98	0.95	0.91	0.88	0.89	0.84	0.91	0.93	0.97
		70	0.99	0.96	0.87	0.86	0.80	0.82	0.92	0.94	0.98

**Fig. 2:**
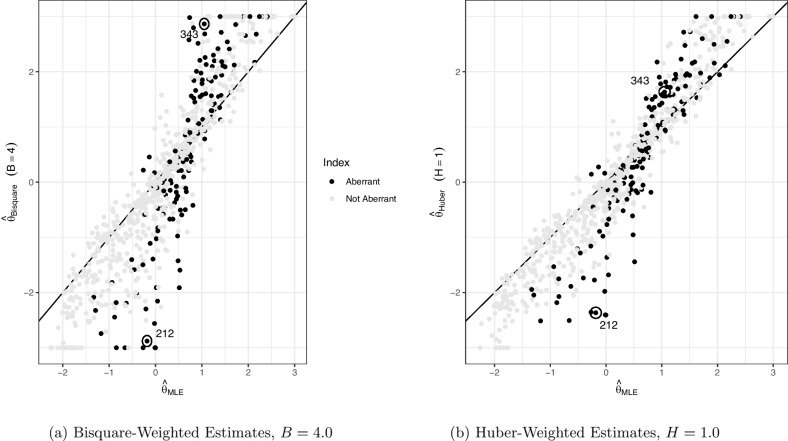
Comparing the MLE of each latent trait with its robust estimate. The figure on the *left* is the bisquare-weighted robust estimate and the figure on the right is the Huber-weighted robust estimate. *Points in black* represent the 20% of subjects with aberrant data, where 33% of their responses are oppositely coded. *Points in gray* represent subjects with “nonaberrant” data. Subjects were not plotted if their MLE or robust estimate did not converge

Tables 5 and 6 display the coverage rates for BRR at severities of 0.1 and 0.3, respectively. Across all conditions, the coverage rates for the robust estimates are greater than those of the MLE. While the coverage rates for the MLE decrease with increasing severity, those for the robust estimate remain relatively similar. Thus, for higher severities, the advantage of the robust estimation on the coverage rates is more prominent, as it is able to mitigate the effects of increased aberrant behavior.

## Applied analysis

We applied our robust estimation procedure to data from the Big Five Inventory-2 (BFI-2) administered to adolescents ages 14 to 17 (Ober et al., 2021). The sample size of $N=838$ contained no missing responses. The scale consists of $J=60$ items on a five-point Likert scale, with 12 items loading onto each of the five personality factors. However, because we are concerned with the unidimensional GRM, we focused on one personality dimension, neuroticism, which is measured by 12 items. Six of these items (50%) were negatively worded such that they should be reverse coded before analysis. Item parameters were estimated using the properly reverse coded data for all 838 subjects.

160 respondents were randomly chosen to be aberrant (20% prevalence). For these subjects, aberrant responses were created such that four negatively worded items had responses opposite to the true intended responses (33% severity). In doing so, we tried to mimic a scenario where subjects were being inattentive and overlooked the negative wording, for example, missing a “NOT” in the question. Discrepancies between the MLE ($\hat{\theta }_{MLE}$) and robust estimates ($\hat{\theta }_{B}$ when the bisquare weight is used and $\hat{\theta }_{H}$ when the Huber weight is used) were compared between the aberrant and the nonaberrant, or undisturbed, responders. It is important to note that there may be other response disturbances that occur in the data (e.g., other types of careless responding), because they frequently plague real data. Therefore, our “nonaberrant” group data may not be entirely undisturbed. However, it is still presumably much less undisturbed than our artificially aberrant data. Likewise, the aberrant group may contain additional response disturbances beyond those artificially created.

Focusing first on the nonaberrant group in Fig. 2 (gray data points), the robust estimates are approximately the same as the MLEs for both the Huber- and bisquare-weighted cases, as most gray points fall close to the identity line, especially for the Huber-weighted cases. However, when we consider the aberrant respondents (black points), a different pattern emerges. For aberrant respondents with small $\hat{\theta }_{ML}$, the corresponding $\hat{\theta }_H$ and $\hat{\theta }_B$ tend to fall below the identity line; whereas aberrant respondents whose $\hat{\theta }_{ML}$ are large tend to have $\hat{\theta }_H$ and $\hat{\theta }_B$ that fall above the identity line. In other words, the robust estimate for a subject tends to be pulled to the extremes (e.g., -2.0 or 2.0), while their MLE tends to be approximately average (i.e., near 0.0). We expect to see this pattern when a respondent misses some negatively worded items, as the mix of some high responses with some low responses will bias the MLE towards 0.

**Table 7:** ML, Huber-weighted, and bisquare-weighted estimates ($\hat{\theta }_{MLE}$, $\hat{\theta }_H$, and $\hat{\theta }_B$, respectively) of neuroticism for three subjects alongside their responses. Note: Aberrant (mistreated reverse coded) items are marked with “*R*”. Values marked with “${-}$” did not converge

ID	$\hat{\theta }_{MLE}$	$\hat{\theta }_H$	$\hat{\theta }_B$	1R	2R	3	4	5R	6R	7	8	9	10	11	12
212	−0.18	−2.37	−2.88	5	5	1	2	5	5	2	1	1	4	2	1
343	1.05	1.63	2.87	2	4	4	4	2	2	5	5	2	2	5	5
650	−	2.85	3.00	1	4	4	5	2	1	5	5	4	5	5	5

Table 7 provides the $\hat{\theta }$s for three example aberrant subjects based on their responses for the 12 items labeled 1 through 12, with “R” indicating one of the four negatively worded items that a respondent might miss. Consider, for example, the response vector for Subject 212. Their responses to regular items mostly consist of 1s and 2s, indicating low neuroticism. Neglecting negatively worded items, their responses to these items remained low, resulting in 5s on these items after reverse coding, which misled the MLE to produce higher estimates. The MLE is biased towards zero, as the aberrant items are increasing the score: a mix of high and low responses leads to an average trait estimate. However, when robust estimation is employed, the trait estimate is much lower ($\hat{\theta }_{H} = -2.37$ and $\hat{\theta }_{B} = -2.88$), indicative of the subject’s actual low neuroticism. This estimate more closely reflects the subject’s true latent trait as suggested by the undisturbed responses. The discrepancy between the robust estimates and the MLE is much greater for Subject 212 in Fig. 2a and b than the non-aberrant subjects in this sample, as it falls further away from the identity line.

Likewise, the response vector for Subject 343 in Table 7 consists of mostly 4s and 5s, indicating a high level of neuroticism. The aberrant responses on the four negatively worded items then wrongfully indicate a lower level of neuroticism, and reduce the MLE. However, the robust procedure properly recovers the trait estimate to the high level of neuroticism ($\hat{\theta }_{H} = 1.63$ and $\hat{\theta }_{B} = 2.87$). Subject 343 falls above the identity line in Fig. 2a and b, demonstrating how their robust estimate increased from the MLE.

Moreover, the trait estimates for Subject 650 demonstrate a common phenomenon in this robust estimation. Oftentimes, the MLE for a subject will not converge while the robust estimates will converge. The robust estimates ($\hat{\theta }_{H} = 2.85$ and $\hat{\theta }_{B} = 3.00$) then reflect the high ability as suggested by the undisturbed responses. Thus, robust estimation can be used to overcome nonconverging MLEs. Because the MLE did not converge, this subject was neither plotted in Fig. 2a nor Fig. 2b. Note that $\hat{\theta }_{B}$ did converge to a value greater than 3.00, so the estimate was truncated to 3.00.

## Discussion

We have proposed a general robust estimation procedure for estimating latent traits with the GRM. We have found that the robust estimation proposed in this paper succeeds in reducing bias for data with various types of disturbances, without substantially increasing the standard error. The estimation is effective at various test lengths and number of response categories.

Both the Huber and bisquare weight functions are effective in reducing the bias in most cases, while providing the same or comparable standard error with respect to the MLE. The choice of either weighting mechanism is left to the researcher. Schuster and Yuan (2011) suggest that the Huber weight should be used over the bisquare weight in cases where nonconvergence may be an issue. Potential nonconverging cases may be identified by examining the data for response vectors which contain all, or predominantly all, responses in the same response category. However, given no concern about nonconvergence, the bisquare weight should be used if reduced bias is preferred. Indeed, in our simulations, we find that bias tends to be slightly reduced for the bisquare weight over the Huber weight, except in some conditions.

Although most differences between the results of the Huber and bisquare weight mechanisms are trivial, the decision of using one over the other may also be influenced by some observable characteristics of the data (e.g., short test lengths and/or few response categories). For data contaminated with a small proportion of aberrant responses (i.e., low severity), in cases of smaller *K*, the bisquare weight function may lead to a greater degree of overcorrection. In this case, the Huber weight function may be preferred. This discrepancy is negligible for greater *K*, where the bisquare weight function tends to reduce the bias more than the Huber weight function. Typically, the bisquare and Huber weights tend to produce very similar average standard errors. However, the Huber weight tends to give a smaller standard error than the bisquare weight for short test lengths ($J=10$), especially for more extreme traits (e.g., $\theta = \pm 2.0, \theta = \pm 1.5$, etc.).

Although we recommend using the tuning parameters $H=1.0$ and $B=4.0$ for the Huber and bisquare weight functions, respectively, it is ultimately up to the researcher to choose a tuning parameter. For a greater degree of downweighting, the tuning parameter can be decreased, while a lesser degree of downweighting can be accomplished with a greater tuning parameter. When the number of categories is low (e.g., $K=3$), the robust estimation is more sensitive to the choice of tuning parameters. For high degrees of aberrant behavior (e.g., 30% severity), using $H=1.0$ and $B=4.0$ operates well; however, for lesser degrees of aberrant behavior (e.g., 10% severity), a greater tuning parameter such as $H=2.0$ and $B=8.0$ may be more optimal in order to avoid overcorrection when items have few categories. However, in our preliminary analysis of *H* and *B*, we found that, generally, $H=1.0$ and $B=4.0$ are the most robust across different test conditions and response behaviors. These values are consistent with the recommendations in the literature as well. Therefore, they are suggested as a rule of thumb.

While the study has demonstrated the advantage of the robust estimator for polytomous items, it can be extended in multiple ways. First, the study is limited to the specific type of residual and weight functions we chose. Different residuals and weighting mechanisms with the same objectives of detecting misfit and downweighting large misfit can be substituted. Future studies may compare this robust estimation with other weighting mechanisms that target aberrant responding, such as those proposed by Wise et al. (2023). In addition, it may be of interest to compare the robust estimation in this analysis to the WLE instead of the MLE, particularly when the test is short, such as ten items (Warm, 1989). Although bias due to aberrant responding is typically more severe, the MLE may contain bias due to short test lengths, and the WLE may be a stronger benchmark in such conditions.

Second, this study is limited by the assumption that the item parameters are known. This assumption is practical given that many IRT-based testing programs use item parameters calibrated from pretesting. However, the true item parameters are never exactly known and are not exempt from misestimations due to aberrant data in the calibration sample (Patton et al., 2019; Tsutakawa & Johnson, 1990). Future studies should investigate these limitations with regards to robust estimation.

Lastly, the analyses in this study are limited to unidimensional data. Future investigations with this estimator could extend to multidimensional polytomous models, such as the multidimensional graded response model (Muraki & Carlson, 1995). It is also important to consider mixed-format assessments, where dichotomous and polytomous items both exist (Hong et al., 2021). A general solution that subsumes multiple item types and underlying latent dimensions will be very desirable. Moreover, robust estimation has been applied to improve estimates of subjects’ working speed under the response time model (Hong et al., 2021), and both item responses and response times information have been used in person fit analysis (Gorney et al., 2024) and detection of rapid guessing behavior (Lu et al., 2020). It is therefore potentially plausible and useful to develop robust estimators for joint models of item responses and response times.

## Data Availability

The example data in the applied data analysis can be found at https://osf.io/fmz74 with additional details on the page https://osf.io/j2s73/.

## Code availability

The materials and code for the simulation study and applied data analysis are available at https://osf.io/jku4d/. The study was not preregistered.
